# Photons Induce Vesicular Exocytotic Release of Glutamate in a Power-Dependent Way

**DOI:** 10.3390/ijms241310977

**Published:** 2023-07-01

**Authors:** Chiara Cervetto, Andrea Amaroli, Sarah Amato, Elena Gatta, Alberto Diaspro, Guido Maura, Antonio Signore, Stefano Benedicenti, Manuela Marcoli

**Affiliations:** 1 Department of Pharmacy, Section of Pharmacology and Toxicology, University of Genova, Viale Cembrano 4, 16148 Genova, Italy; chiara.cervetto@unige.it (C.C.); sarah.amato@edu.unige.it (S.A.); guido.maura@unige.it (G.M.); 2 Interuniversity Center for the Promotion of the 3Rs Principles in Teaching and Research (Centro 3R), 56122 Pisa, Italy; 3 Department of Earth, Environment and Life Sciences, University of Genova, Viale Benedetto XV 5, 16132 Genova, Italy; 4 DIFILAB, Department of Physics, University of Genova, Via Dodecaneso 33, 16146 Genova, Italy; elena.gatta@unige.it (E.G.); diaspro@fisica.unige.it (A.D.); 5 Nanoscopy, Nanophysics, Istituto Italiano di Tecnologia-IIT, Via Morego 30, 16133 Genova, Italy; 6 Biophysics Institute, National Research Council-CNR, Via de Marini, 6, 16149 Genova, Italy; 7 Therapeutic Dentistry Department, Institute of Dentistry, Sechenov First Moscow State Medical University, Trubetskaya Str. 8, b. 2, 119992 Moskow, Russia; dr.signore@icloud.com; 8 Department of Surgical Sciences and Integrated Diagnostics, University of Genova, Viale Benedetto XV 6, 16132 Genova, Italy; stefano.benedicenti@unige.it; 9 Center of Excellence for Biomedical Research, University of Genova, 16132 Genova, Italy

**Keywords:** laser therapy, photobiomodulation, LLLT, light therapy, nerve terminals, exocytotic glutamate release, sodium and calcium channels, sodium channels, mouse cerebral cortex

## Abstract

Increasing evidence indicates that photobiomodulation, based on tissue irradiation with photons in the red to near-infrared spectrum, may be an effective therapeutic approach to central nervous system disorders. Although nervous system functionality has been shown to be affected by photons in animal models, as well as in preliminary evidence in healthy subjects or in patients with neuropsychiatric disorders, the mechanisms involved in the photobiomodulation effects have not yet been clarified. We previously observed that photobiomodulation could stimulate glutamate release. Here, we investigate mechanisms potentially involved in the glutamate-releasing effect of photons from adult mouse cerebrocortical nerve terminals. We report evidence of photon ability to induce an exocytotic vesicular release of glutamate from the terminals of glutamatergic neurons in a power-dependent way. It can be hypothesized that photobiomodulation, depending on the potency, can release glutamate in a potentially neurotoxic or physiological range.

## 1. Introduction

Increasing evidence indicates that photobiomodulation (PBM), previously known as low-level laser/light therapy (LLLT) and based on the irradiation of tissue with photons in the red to near-infrared spectrum, may be an effective therapeutic approach to central nervous system (CNS) disorders [1,2,3,4]. PBM has been shown to promote axon growth and nerve regeneration [5,6], to attenuate ischemia neurotoxicity in in vitro models [7], to rescue rodent neurons from neurotoxic injury and to reduce long-term deficits after stroke or traumatic brain injury in animal models ([5]; see also [8]). In addition, it has been reported to reduce amyloid-beta neuropathology in an Alzheimer’s disease model [9] and to enhance memory and improve cognitive deficits in animal models [5,10]. PBM has also been proposed to possess neuroregenerative potential [5]; the mechanisms responsible for the effect seem to involve the anti-inflammatory or antioxidant properties of light and the improvement of angiogenesis and microcirculation [3,11,12]. Preliminary clinical evidence reports that transcranial PBM could induce cognitive enhancement and improve working memory and attention in healthy adults [13]. Additionally, intranasal or transcranial PBM therapy has been proposed for neurological and neuropsychiatric disorders: it was reported to support cognitive and memory functions in Alzheimer’s disease patients, to improve cognition and motor function in Parkinson’s disease, to promote recovery from traumatic or ischemic brain injury and to improve mood disorders including depression (see [2,4,8,14,15,16] and references therein). Nevertheless, placebo-controlled randomized clinical trials are warranted to understand the role of PBM and the optimal treatment parameters in the management of neuropsychiatric disorders and neurodegenerative diseases (see [4,16]).

Although CNS function has been repeatedly shown to be affected by photons in animal in vitro or in vivo models, as well as in preliminary clinical evidence in healthy subjects or in patients with neuropsychiatric disorders, the mechanisms involved in the PBM effects have not yet been fully clarified. PBM is based on primordial photoacceptive properties of cell molecules which are photo-energized during the irradiation of tissue with light from the visible to the near-infrared electromagnetic spectrum [17]. Consistent evidence indicates that mitochondria and, more specifically, cytochrome c oxidase may be PBM targets; mitochondria being targeted might involve the ability of PBM to affect CNS functioning ([18]; see also [19]). In fact, the primary cellular targets of photons, cytochromes belonging to the mitochondria respiratory chain, were described as being modulated according to wavelengths and light therapy parameters [18]. Particularly, the 810 nm wavelength has a main effect on mitochondrial complex IV and a lower effect on complex III, while complexes I and II are not affected [20]. The leading hypothesis is that increased electron transport and variation in the membrane potential of the photo-energized mitochondria result in improved production of ATP [18,20,21]. In addition, it has been described that flavin and opsin can interact with visible light [22,23], that latent transforming growth factor-β1 can be modulated by 810 nm laser light [24], and that S-nitrosylated protein can be dissociated by visible and near-infrared light [12]. Moreover, light-sensitive ion channels have been hypothesized to be activated by PBM, allowing Ca^2+^ entry into the cells [18,20]. Then, as secondary targets, activation of signaling pathways with consequent activation of transcription factors and increased expression of genes related to protein synthesis, cell migration and proliferation, anti-inflammatory signaling, anti-apoptotic proteins and antioxidant enzymes could also contribute to the PBM effects in CNS [21,22,23,24].

Despite PBM effectiveness in animal models and in clinical studies, the issue of the poor understanding of the mechanisms of action of PBM in CNS remains unsolved. To better understand these mechanisms, we focused on analyzing the ability of photons to evoke a glutamate release, as glutamate is a key neurotransmitter involved in healthy brain function and, conversely, dysfunction [25,26]. In fact, we previously observed that near-infrared PBM stimulated cerebrocortical glutamatergic neurons to release great amounts of glutamate [27]. Here, we investigated the mechanisms involved in the glutamate-releasing effect of PBM and found evidence that PBM (irradiated with a flat-top hand-piece in continuous wave, 810 nm, 60 J/cm^2^, 1 W, 1 W/cm^2^, 60 s) could stimulate the exocytotic vesicular release of glutamate from nerve terminals (synaptosomes) acutely isolated from the adult mouse cerebral cortex. We also assessed the effect of an 810 nm PBM at the lower parameter of 6 J/cm^2^, 0.1 W, 0.1 W/cm^2^, 60 s, and reported that near-infrared light was able to stimulate glutamate release from the nerve terminals in a power-dependent way. The main findings of our study are that PBM facilitates the vesicular glutamate release in a power-dependent way, and it is possible to choose a power evoking a neurotransmitter release similar to that evoked by a quasi-physiological stimulation of the nerve terminals. It can be surmised that keeping the delivered area surface (1 cm^2^) and the time of irradiation (60 s) constant, PBM can release glutamate in a potentially neurotoxic or physiological range based on the dose administered. Although the precise target(s) of PBM in the nerve terminals and the processes involved require further investigation, our study can contribute to a better understanding of the mechanisms of action of PBM in CNS. In fact, evidence of vesicular glutamate release facilitation, and therefore of the release mode specific for neurons at a synaptic level and selectively responsible for intercellular communication through neurotransmitters in CNS, could help to clarify the mechanisms involved in PBM effectiveness in healthy brain function as well as in brain dysfunction.

## 2. Results

### 2.1. Synaptosomes Proved to Be a Purified Preparation of Nerve Terminals

**Figure 1:**
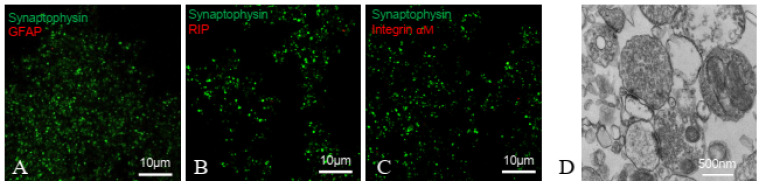
Cortical synaptosomes: immunofluorescent and ultrastructural analysis. (**A**–**C**). Immunofluorescent analysis. To assess the contamination by glial particles, cortical synaptosomes were double labelled with primary antibodies for synaptophysin (**A**–**C**) and for the astrocyte marker glial fibrillary acid protein GFAP (**A**), the oligodendrocyte marker RIP (**B**), or the microglia marker integrin-αM (**C**). Representative merge images are shown. (**D**) Electron microscopy analysis. A representative image of cortical synaptosomes is shown. The scale bars are indicated in the panels.

Immunofluorescent confocal microscopy analysis of synaptosomes showed that they were positive for the nerve terminal marker synaptophysin, and negative for the astrocytic marker glial fibrillary acidic protein (GFAP), the microglial marker integrin-αM and the oligodendroglial marker RIP (Figure 1A–C). This clearly indicates that synaptosomes are a purified preparation of nerve terminals, negligibly contaminated by astroglial, oligodendrocyte or microglial particles. Ultrastructural analysis confirmed that synaptosomes possess a nerve terminal main feature: single synaptosomes appear equipped with synaptic vesicles docked at the presynaptic active zones lying at the interface between the presynaptic nerve terminal and the synaptic cleft; in some cases, opposed pre- and postsynaptic specializations containing electron-dense material were maintained in the preparation (Figure 1D). Notably, in some synaptosome sections, mitochondria structures were identified.

### 2.2. Release of Glutamate from Synaptosomes: Effect of Laser Light

**Figure 2:**
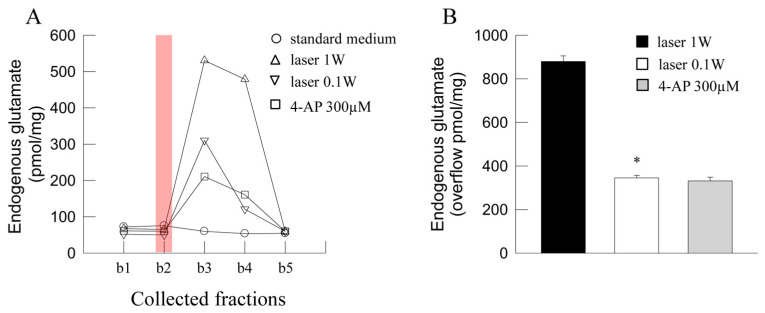
Photon effect on glutamate release from mouse cerebrocortical nerve terminals. (**A**) Representative time-course of the endogenous glutamate release from nerve terminals in control conditions and in response to laser light (60 J/cm^2^, 1 W, 60 s) or (6 J/cm^2^, 0.1 W, 60 s) is shown. 4-Aminopyridine (4-AP) was added (3 min) to the medium during superfusion, starting 1 min before the second collected fraction b2. Light was applied (6 or 60 J/cm^2^, 0.1 or 1 W, 60 s; vertical red bar) during superfusion; other experimental details in Materials and Methods. (**B**) Bars represent the glutamate overflow when synaptosomes were exposed to laser light (60 J/cm^2^, 1 W, 60 s, black bar; 6 J/cm^2^, 0.1 W, 60 s, open bar), or to 4-AP (300 mM, 3 min, gray bar) during superfusion. Data are means ± SEM (bars) of 3–7 experiments on different days. * *p* < 0.05 compared with the response to 60 J/cm^2^, 1 W, 60 s; one-tailed Mann–Whitney test.

The efflux of endogenous glutamate in the first two basal fractions collected from superfused synaptosomes amounted to 79.83 *±* 5.98 pmol/mg prot x min (*n* = 18). Exposure of synaptosomes to laser light (60 J/cm^2^, 1 W, 1 W/cm^2^, 60 s, continuous wave) evoked a great glutamate overflow, while the overflow evoked by a 0.1 W laser light (6 J/cm^2^, 0.1 W, 0.1 W/cm^2^, 60 s) appeared significantly lower (Figure 2). Exposure to 4-Aminopyridine (4-AP) evoked a glutamate overflow significantly lower than the release evoked by a 1 W laser light, while not significantly different from the overflow evoked by a 0.1 W laser light (Figure 2).

**Figure 3:**
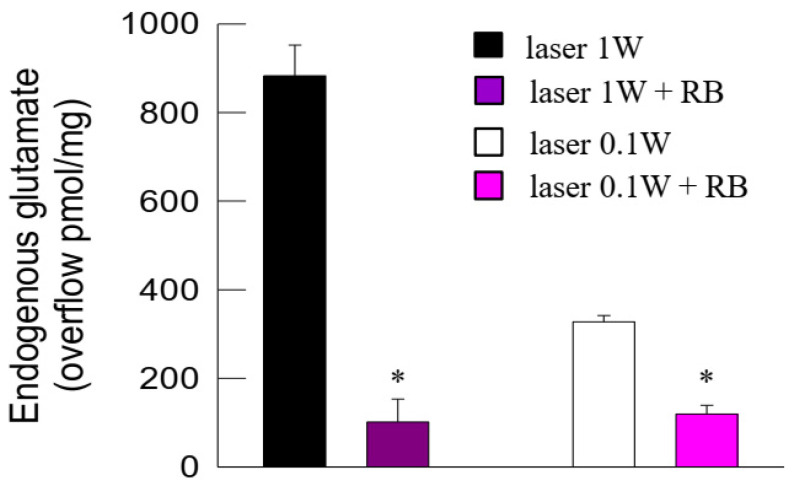
Photon effect on glutamate release from mouse cerebrocortical nerve terminals: inhibition of the photon-evoked glutamate release by the vesicular glutamate transporter (VGLUT) inhibitor Rose Bengal (RB). Bars represent the endogenous glutamate overflow when synaptosomes in control conditions (black or open bars) or in the presence of RB (purple or dark purple bars) were exposed to light (60 s). Light was applied (6 or 60 J/cm^2^, 0.1 or 1 W, 60 s) during superfusion; other experimental details in Materials and Methods. Data are means ± SEM (bars) of three experiments on different days. * *p* < 0.05 compared with laser in the absence of RB; one-tailed Mann–Whitney test.

The efflux of endogenous glutamate in the first two basal fractions collected from superfused synaptosomes pre-incubated with the vesicular glutamate transporter (VGLUT) inhibitor Rose Bengal (RB) amounted to 73.91 *±* 11.0 pmol/mg prot/min (*n* = 8), which was not significantly different from the basal release in control conditions. The glutamate overflow from synaptosomes evoked from 1 W or 0.1 W laser light was inhibited in the presence of RB (Figure 3).

**Figure 4:**
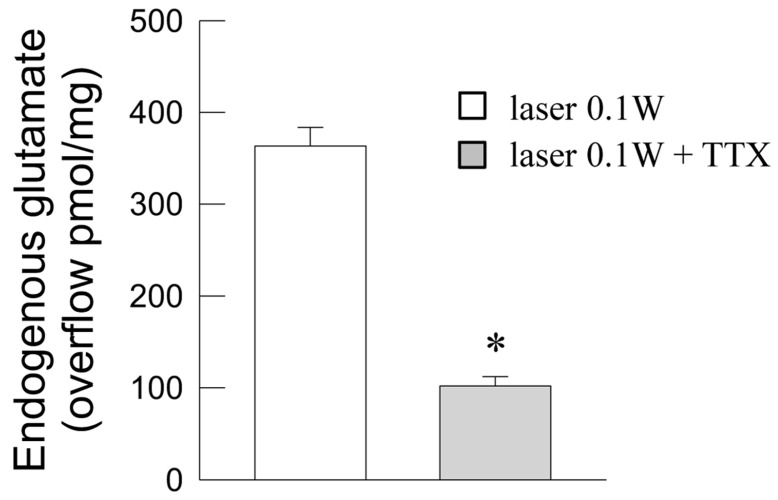
Photon effect on glutamate release from mouse cerebrocortical nerve terminals: inhibition of the photon-evoked glutamate release by the Na^+^ channel blocker TTX. Bars represent increase in endogenous glutamate overflow when synaptosomes (in control conditions (open bar) or in the presence of TTX (gray bar)) were exposed to light (60 s). Light was applied (6 J/cm^2^, 0.1 W, 60 s) during superfusion; other experimental details in Materials and Methods. Data are means ± SEM (bars) of four experiments on different days. * *p* < 0.05 compared with laser in the absence of TTX; one-tailed Mann–Whitney test.

The ability of 0.1 W laser light to evoke glutamate overflow from synaptosomes was further investigated. The glutamate overflow was markedly inhibited in the presence of the blocker of voltage-dependent Na^+^ channels tetrodotoxin (TTX) (Figure 4).

**Figure 5:**
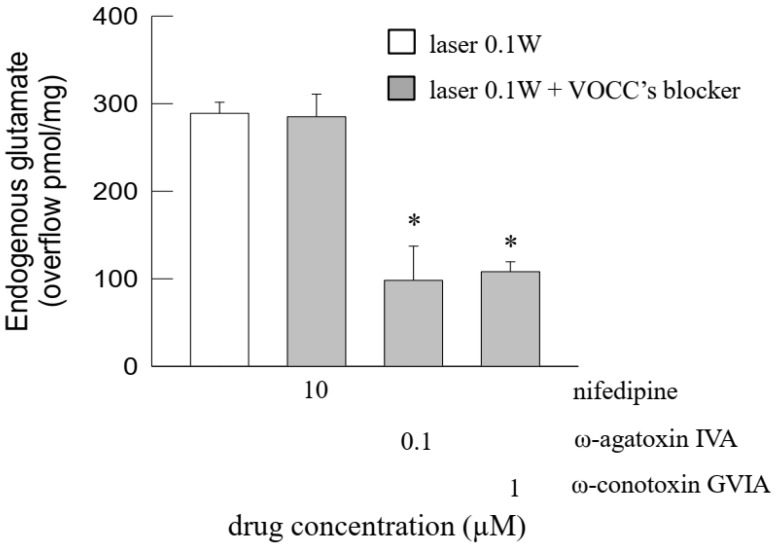
Photon effect on glutamate release from mouse cerebrocortical nerve terminals: inhibition of the photon-evoked glutamate release by blockers of voltage-dependent Ca^2+^ channels (VOCCs). Bars represent the endogenous glutamate overflow when synaptosomes were exposed to light (60 s), in the absence (open bar) or in the presence (gray bars) of drugs at the concentrations indicated. Light was applied (6 J/cm^2^, 0.1 W, 60 s) during superfusion; other experimental details in Materials and Methods. Data are means ± SEM (bars) of 3–7 experiments on different days. * *p* < 0.05 vs. laser in the absence of drugs; Kruskal–Wallis analysis and multiple comparison test vs. control condition (laser 0.1 W).

Furthermore, the light-evoked release of glutamate was greatly reduced by the blockers of the voltage-dependent Ca^2+^ channels of the P/Q type ω-agatoxin IVA or of the N type ω-conotoxin GVIA, while the L-type Ca^2+^ channel blocker nifedipine was ineffective (Figure 5).

### 2.3. Power Evaluation and Temperature Measurement

**Figure 6:**
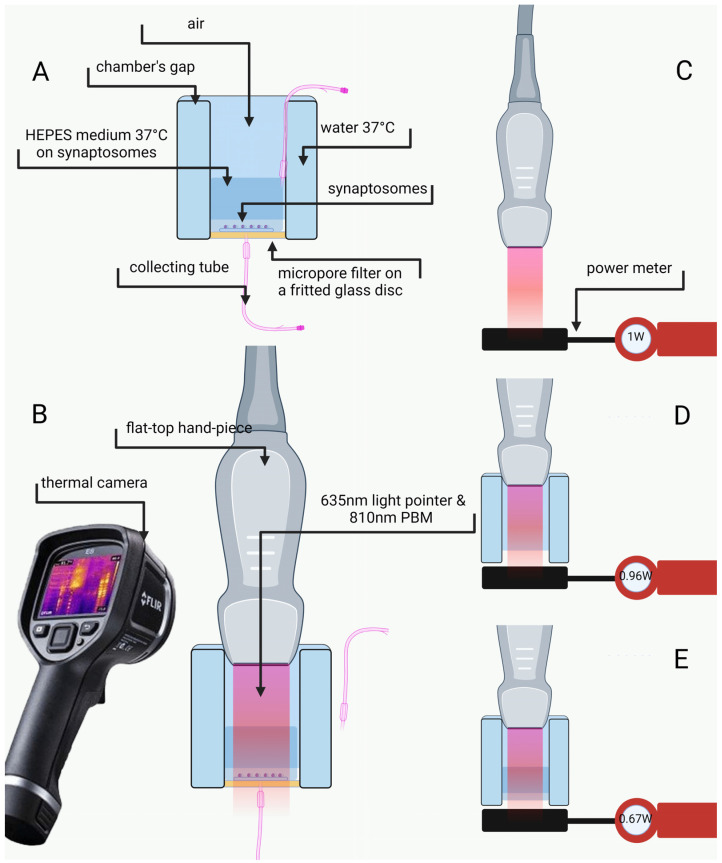
Experimental setup. Brief description of the superfusion chamber (**A**). Synaptosomes irradiation and temperature monitoring (**B**). Evaluation of the power through the power meter (**C**–**E**). Irradiation without the chamber (**C**). Irradiation in the chamber without HEPES medium (**D**). Irradiation with HEPES medium, like in the experimental setup (**E**). Other experimental details in Materials and Methods.

The distance between the flat-top hand-piece delivering photons and the synaptosomes on the chamber bottom did not affect the power delivered, indicating that air does not affect the transmission of photons (see Figure 6). Indeed, 1 W set in the device corresponded to 0.96 ± 0.04 W measured by the power meter (see the Figure 6C,D) at the chamber bottom. Conversely, about 30% of the power was absorbed by the superfusion medium (1 cm wet film thickness). The synaptosomes, therefore, were reached by 0.67 ± 0.06 W or 0.065 ± 0.007 W when PBM 1 W or 0.1 W, respectively, was applied (see the Figure 6E). No temperature change was observed during irradiation.

## 3. Discussion

Investigation on the effects of photons on glutamatergic transmission was carried out on nerve terminals (synaptosomes) acutely prepared from adult mouse cerebral cortex. We here demonstrate that synaptosomes represent a purified preparation of nerve terminals, not contaminated by astrocytic, microglial or oligodendrocyte particles. Furthermore, mitochondria were observed in the synaptosomes; notably, mitochondrial function was reported to be preserved in isolated cerebrocortical nerve terminals [28]. Electron microscopy also indicated that the isolated nerve terminals retained the hallmarks of mammalian presynaptic nerve terminals in situ [29,30] or in cultured neurons [31] as they were filled with vesicles exhibiting the typical appearance of the small clear synaptic vesicles for storage of the classical neurotransmitters (diameter about 30 nm; see [32,33]) clustered at the active zones and docked for fusion and transmitter release. In fact, the nerve terminals prepared from CNS regions have been repeatedly shown to release glutamate in a vesicular way upon stimulation (see [34], and references therein). We report here that an 810 nm PBM evoked a vesicular exocytotic glutamate release from the nerve terminals, involving activation of the plasma membrane Na^+^ channels and opening of Ca^2+^ channels. Of note, the amount of photon-evoked glutamate release appears dependent on the delivered PBM power. The investigation, taking advantage of the specific features of the in vitro model and assessing the direct action of chemical or physical stimuli upon the nerve terminals, has allowed us to conclude that photons can evoke a release of glutamate vesicularly, a typical modality by which the neurotransmitter is released from neurons at synapses. Mechanistically speaking, the study adds information that can help to clarify the mechanisms of action for the reported PBM effects on CNS in vivo, thus helping hypothesize possible mechanisms for antidepressant, cognitive and memory effects (see below).

The main limitation of this study lies in the fact that the observations were obtained from healthy animals in an in vitro model. However, it is to be considered that proper in vitro models can aid in the understanding of PBM cell targets and the mechanisms involved in the PBM effects in CNS. Therefore, this investigation is a preliminary step to translate the study on pathological in vitro and in vivo models, e.g., on models of epilepsy or neuropsychiatric disorders. In fact, laser light performance in therapeutic applications is highly related to the tissue’s material properties such as diffusivity, reflectivity and thermal conductivity [35]. Thus, studying the correct laser parameters for the replication of the therapeutic application in vivo will be necessary. Furthermore, for a comprehensive view of PBM effects in CNS, an assessment of the effects on the release of other neurotransmitters in addition to glutamate would be required. These points could be addressed in future investigations.

### 3.1. Photon-Evoked Glutamate Release from Glutamatergic Nerve Terminals in a Dose-Dependent Way

PBM 1 W evoked a great amount of glutamate release from the nerve terminals. The amount was greater than that released when the glutamatergic nerve terminals were exposed to physiological stimuli, such as depolarization with 4-Aminopyridine (4-AP). Indeed, the blocker of voltage-sensitive K^+^ channels 4-AP, mimicking the physiological mechanisms of terminal depolarization and producing a Ca^2+^-dependent release of neurotransmitters ([36]; see also [37] and references therein), is considered a quasi-physiological stimulus to investigate the characteristics of the evoked release of glutamate. While the huge amount of glutamate released by 1 W PBM might allow glutamate to reach potentially neurotoxic extracellular levels, keeping the delivered area surface (1 cm^2^) and the time of irradiation (60 s) constant, PBM at lower power (0.1 W) evoked a release of glutamate that was comparable to the release evoked by the quasi-physiological stimulus 4-AP.

The ability of photons to evoke glutamate release from the nerve terminals is of special interest considering that glutamate is the primary excitatory neurotransmitter in mammal CNS. The glutamate extracellular concentrations at the synapses result from the balance between the glutamate release from glutamatergic nerve terminals or astrocyte processes and the glutamate clearance through uptake from the nerve terminals and astrocyte processes [26]. In fact, the glutamate extracellular levels are tightly regulated in a healthy brain [26]. Indeed, glutamate and glutamate receptor activation subserve crucial physiological functions that play roles in cell proliferation, migration, differentiation and plasticity during development [38], as well as in synapse plasticity, learning and memory and cognitive functions in the adult brain [25]. Conversely, dysregulation of glutamatergic transmission is a common feature in a diseased brain and is recognized to be involved in CNS pathological conditions including neurodegenerative/neuroinflammatory diseases (e.g., Alzheimer’s or Parkinson’s disease or schizophrenia; [39,40,41,42]), as well as in mood disorders including depression [43]. Accordingly, glutamate has long been considered a therapeutic target in neuropsychiatric disorders [44,45]. Our findings indicate that PBM could activate the glutamatergic transmission in CNS with effects ranging from facilitation of physiological roles of glutamate transmission to potential neurotoxicity, depending on the power. Although the significance of the PBM-evoked glutamate release is worth further investigation, the findings might contribute to clarify possible mechanisms of the PBM therapeutic properties in CNS disorders. We propose that glutamate transmission-enhancing effects of PBM might contribute to the reported effects against depression or cognitive impairment.

Notably, PBM has been proposed to possess antidepressant effects in patients [14,15]. It is of note that approaches to glutamatergic transmission are under investigation for antidepressant treatment [43,46]. Rapid-acting antidepressants (ketamine and psychedelic agents) were reported to disinhibit glutamate transmission, increase extracellular glutamate and synapse numbers, and reverse synaptic deficits caused by chronic stress leading to a rapid and robust antidepressant therapeutic response even in treatment-resistant patients [47,48]. Fast changes in synaptic function and plasticity were demonstrated with the restoration of spine density within hours of treatment with rapid-acting antidepressants, and it is hypothesized that induction of synaptogenesis reverses the depression-associated loss of synaptic connectivity and restores cognitive and emotional function [48]. The Food and Drug Administration has recently approved the first oral *N*-methyl *D*-aspartate (NMDA) receptor antagonist for major depression treatment [49]. However, unanswered questions remain regarding efficacy, safety, implementation and best practices related to the use of rapid-acting antidepressants in patients [50]. PBM could be investigated as a safer glutamate-enhancing therapeutic alternative.

Furthermore, the PBM-evoked glutamate release might be relevant to clarify the mechanisms by which PBM can possess memory improvement capacity in both healthy individuals [13] and individuals with pathological cognitive decline including dementia and Alzheimer’s disease (AD) ([51]; see [16] and references therein). The role of glutamate and glutamate receptor involvement in synapse plasticity, learning and memory is well established (see [39,52]). Age-related changes in glutamatergic transmission have been described in the hippocampus, in the prefrontal cortex and in motor and sensory areas helping to explain the subsequent cognitive, motor and sensory decline in healthy aging individuals [53]. Similarly to what has been observed in humans, rats and mice show a subtle decrease in glutamatergic synapses and neurons during aging; animal models of successful aging may suggest that healthy cognitive aging requires maintenance of glutamatergic signaling throughout the aging process [53]. Notably, glutamatergic transmission dysregulation has been proposed to signal the transition from physiological to pathological aging, including dementia and Alzheimer’s disease [53]. Pathological accumulation of glutamate in AD can induce neurotoxicity due to time-related exposure, over-stimulating post-synaptic response and Ca^2+^ entry into neurons [54,55]. It is suggested that while synaptic NMDA receptor signaling is required for the survival of neurons, extrasynaptic NMDA receptor signaling activated by the spillover of astrocyte- or presynaptic terminal-released glutamate can antagonize the synaptic pro-survival signaling pathway and tilt the balance toward excitotoxicity and ultimate neurodegeneration. The hypothesis is supported by the beneficial effects of memantine which suppresses the extrasynaptic NMDA receptor signal in moderate-to-severe AD [39]. As far as the potential usefulness of PBM in AD (see [9,51,56]) is concerned, it is of note that the glutamatergic neuron activity is compromised in AD due to synapse destruction and neuron death, and its deficit can influence memory, cognition and behavior, including cortical and hippocampal processing functions [57,58]. It can be surmised that the glutamate-releasing effect of PBM from glutamatergic nerve terminals and its inability to stimulate the release of glutamate from the astrocyte processes [27] could facilitate synaptic vs. non-synaptic transmission, therefore favoring synaptic vs. non-synaptic NMDA receptor activation. On the other hand, excessive extracellular levels of glutamate and consequent excessive activation of ionotropic glutamate receptors are responsible for excitotoxicity, a major common pathway for neuron damage in chronic neurodegenerative diseases including AD [59] and in acute brain insults (ischemia, traumatic brain injury [60,61]). In this scenario, it is of note that when neurons were exposed to high extracellular glutamate levels, PBM was able to reduce excitotoxicity [62]. It appears, therefore, that PBM might have dual effects on glutamatergic transmission in the brain: it could evoke synaptic glutamate release (accounting for positive effects on memory, healthy aging and depression, and conceivably on AD), while possibly interfering with mechanisms responsible for neuron damage in chronic or acute excitotoxicity. In fact, PBM was found to increase the ATP levels, to raise mitochondrial membrane potential and to reduce intracellular Ca^2+^ levels, as well as the oxidative stress and nitric oxide production in primary cortical neurons exposed to excitotoxic glutamate levels [62]; these effects abrogating excitotoxicity might explain the beneficial laser light effects in some CNS pathological conditions. Therefore, promising PBM results in AD patients might be related to an effect on synaptic glutamate release mainly found in early disease stages, and later to inhibition of neuroinflammation, preservation of mitochondrial function, and suppression of oxidative damage together with inhibition of amyloid deposition (see [9,23,51,56]).

The power dependency of the glutamate-releasing properties of PBM deserves a comment. Of note, when the effects of an 810 nm PBM at 1 W and 0.1 W on ATP production and Ca^2+^ homeostasis were studied in pre-nervous organisms, such as protozoa, they were able to modulate cell–cell interaction through neurotransmitters [63,64,65,66] and a power-dependent effect on ATP production was described [67]. Therefore, great attention needs to be paid to the power issue to set the optimal PBM treatment parameters in neuropsychiatric disorders to allow for the facilitation of the physiological roles of glutamate transmission and to avoid potential neurotoxicity. In fact, the definition of the irradiation parameters seems to also be a crucial point in the use of PBMs in cancer, where PBM therapy as supportive therapy in cancer, and as a therapy against the side effects of anti-cancer therapy, has been reported to exhibit a biphasic dose–response that warrants optimal tissue-specific irradiation dose parameters to be defined (see [68,69]).

### 3.2. Photons Evoked Vesicular Exocytotic Release of Glutamate Release from the Nerve Terminals

We previously reported that the photon-evoked release of glutamate was dependent on the availability of extracellular Ca^2+^, consistent with activation of vesicular exocytotic release. Vesicular storage and subsequent exocytotic release of neurotransmitters are the key processes of chemical signal transmission [70] and play a central role in intercellular communications in CNS. Notably, clusters of vesicles at synaptic release sites have been shown to be composed of a pool containing synapsin, which is required to sustain the release of neurotransmitters at a high rate [71], and a pool devoid of synapsin and located adjacent to the presynaptic membrane. In mammal CNS, the synapse morphological hallmarks can be observed with an electron microscopy: presynaptic nerve terminals contain abundant synaptic vesicles of approximately 30–40 nm diameter; at the synaptic contacts, the presynaptic plasma membrane is thickened into an active zone where several synaptic vesicles are docked; facing the presynaptic active zone, a thickening in the postsynaptic plasma membrane is referred to as postsynaptic density [72]. Notably, the synapse hallmarks were maintained in our synaptosomal preparation: the nerve terminals appeared equipped with abundant small vesicles and with synaptic vesicles docked at the presynaptic active zones; in some cases, the synaptic cleft and opposed pre- and postsynaptic specializations appeared maintained (see Figure 1D).

The synaptic vesicle exocytosis is the process by which a synaptic vesicle fuses with the plasma membrane of the nerve terminal and releases its contents into the synaptic cleft, allowing for a fast release of signals and a fast signal termination [72,73]. The focal point of the vesicle cycle is a Ca^2+^-triggered exocytosis: the working models of synaptic vesicle exocytosis include synaptotagmin-1 as a key protein which, as a consequence of Ca^2+^ binding, triggers the exocytosis ([74] and references therein).

In this process, specific vesicular transporters are responsible for loading the neurotransmitters into the synaptic vesicles [75]. In particular, vesicular exocytotic release of glutamate requires the activity of VGLUTs to load glutamate into the synaptic vesicles ([76] and references therein). The compound RB is a potent VGLUT inhibitor [77]. In our study, by proving that the photon-evoked release was greatly reduced when the loading of exocytotic vesicles to the neurotransmitter was prevented by RB, we confirmed that photons, both at 1 W and 0.1 W, activated vesicular exocytotic glutamate release.

### 3.3. The Photon-Evoked Glutamate Release Involved Activation of Plasma Membrane Na^+^ Channels and Opening of Ca^2+^ Channels

The mechanisms possibly involved in the ability of photons to evoke exocytotic glutamate release were further investigated. In particular, the engagement of voltage-activated channels at the plasma membrane, primarily involved in the activation of vesicle exocytosis [78], was assessed. Our findings demonstrated the ability of photons to activate both voltage-dependent Na^+^ and voltage-dependent Ca^2+^ channels, as indicated by the effectiveness of the blocker of Na^+^ channels TTX, and of toxins selectively blocking Ca^2+^ channels. As a matter of fact, well-recognized in the process of exocytosis is the sequential intervention of voltage-dependent Na^+^ channels in response to the action potential invading the nerve terminal (allowing membrane depolarization) and then the opening of voltage-dependent Ca^2+^ channels [79]. The Ca^2+^ channels primarily involved in activation of vesicle exocytosis belong to the N-type, located in the microdomain of the active zones near the sites for vesicle fusion, and the P/Q-type in particular as far as the glutamatergic nerve terminals are concerned [80,81,82,83]. Notably, we found that activation of these Ca^2+^ channel types was involved in the glutamate-releasing effect of photons, as indicated by the ability of the N-type Ca^2+^ channels blocker ω-conotoxin GVIA, and of the P/Q-type Ca^2+^ channels blocker ω-agatoxin IVA, to impair it. The L-type Ca^2+^ channels blocker nifedipine was ineffective, and indeed neuronal L-type channels are only marginally involved in the exocytotic release of neurotransmitters ([80]; see [84]). Further investigation would be required to better understand how photons can lead to the opening of the plasma membrane voltage-dependent Na^+^ channels and the processes responsible for channel opening. Nevertheless, we can remember that the ability of PBM to affect the membrane properties also leading to the opening of voltage-dependent channels have already been reported [85,86].

## 4. Materials and Methods

### 4.1. Animals

Mice (males, C57BL/6 J; 8–12 weeks old) were housed at the animal care facility of the Department of Pharmacy (DIFAR), University of Genova, Italy in constant conditions (22 ± 1 °C; 50% humidity; lights on 7 a.m.–7 p.m.), with water ad libitum and free access to a standard diet. Animal care and experimental procedures complied with the Directive 2010/63/EU and with Italian Legislative Decree 26/2014 and were approved by the Italian Ministry of Health (protocol n° 75F11.N.0RF, of November 2021), in accordance with Ministerial Decree 116/1992. Every effort was made to minimize animal suffering and the number of animals used.

### 4.2. Preparation of Purified Nerve Terminals and Perfusion

Purified nerve terminals (synaptosomes) were prepared from mouse cerebral cortex as previously described [87,88,89]. Briefly, after tissue homogenization in Tris-buffered sucrose (pH 7.4) and centrifugation, the supernatant was stratified on a discontinuous Percoll gradient (2, 6, 10, 20% (*v*/*v*) in Tris-buffered sucrose) and centrifuged. Synaptosomes were collected at the 10–20% (*v*/*v*) Percoll layers. Synaptosomes were suspended in standard HEPES medium (mM: NaCl 128, KCl 2.4, MgSO_4_ 1.2, KH_2_PO_4_ 1.2, CaCl_2_ 1.0, HEPES 10, glucose 10, pH 7.4); equal aliquots were then transferred at the bottom of parallel superfusion chambers maintained at 37 °C and superfused, and superfusate fractions were collected in 3 min samples. After 38 min superfusion, synaptosomes were exposed to laser light (6 or 60 J/cm^2^, 0.1 or 1 W, 60 s) or to 4-AP (300 mM; 3 min). The effect of the VGLUT inhibitor RB was assessed when the synaptosomes were preincubated 30 min at 37 °C with 0.5 μM RB [87,90]. Drugs were added 8 min before the laser light when the effect of the Na^+^ channel blocker TTX or of the voltage-dependent Ca^2+^ channels was evaluated. In each experiment, at least one chamber was run as a control for each condition and was not exposed to light. It is to be noted that by this methodological approach we could monitor the release of the neurotransmitter from a synaptosomal monolayer by removing any active substance that might be released and, therefore, avoid indirect effects (see [34]). This approach allowed us to study the direct effects of substances on transmitter release [34,87,89] and also allowed for the study of the direct effects of a physical stimulus such as laser light [27].

### 4.3. Delivering Device and Parameters Setting

**Figure 7:**
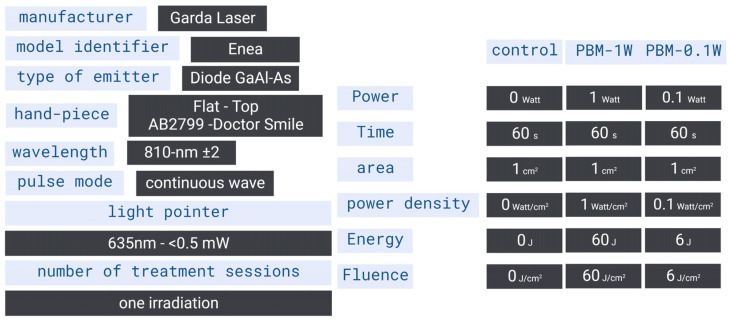
Device specifications and irradiation parameters.

The experimental parameters and delivering instrument specifications are shown in Figure 6 and Figure 7. The synaptosomes were exposed during perfusion to the 810 nm diode laser (GaAlAs) device (Garda Laser, 7024 Negrar, Verona, Italy) equipped with the FT-HP hand-piece AB2799 (Doctor Smile–LAMBDA Spa–Vicenza, Italy). According to our previous studies, the FT-HP is able to irradiate a spot area with a consistent energy distribution independently of the distance [91]. To follow our previous study [27], in an experimental setup, we set the 810 nm device to irradiate 1 W of power in CW mode for 60 s on a circular spot of 1 cm^2^. These parameters allowed for the generation of a power density of 1 W/cm^2^ and a fluence (dose) of 60 J/cm^2^ (energy administered = 60 J) (PBM-1W). A second experimental setup was scheduled at a setting of 810 nm, 0.1 W, CW mode for 60 s and a circular spot of 1 cm^2^ to generate 0.1 W/cm^2^, 6 J/cm^2^ and 6 J (PBM-0.1 W). A 635 nm light pointer (negligible power, <0.5 mW) was used to visualize the exposed area in both treatments. Control was performed using the 635 nm light pointer but with the device set to irradiate 0 W and 0 J for 60 s.

### 4.4. Irradiation Design, Laser Power Assessment and Temperature Monitoring

Despite the consistency of the FT-HP irradiation [91], the experimental setup can influence the effective power arriving at the synaptosomes target. As shown in Figure 6, the FT-HP was fixed at the top of the superfusion chamber (maintained at 37 °C), while the synaptosomes were at the bottom. To arrive at the synaptosomes, photons cover a way across air and physiological medium that could influence the actual power delivered to the nerve terminals. Additionally, temperature (although unlikely) could increase during irradiation. Therefore, the monitoring setup shown in Figure 6 was designed. For this purpose, the PM160T-HP power meter (dynamic range: 0.01 W–70 W; ThorLabs, Bergkirchen, Germany) and the FLIR ONE Pro-iOS thermal camera (dynamic range: −20 °C/+400 °C; resolution 0.1 °C; FLIR Systems, Inc. designs, Portland, OR, USA) were employed.

### 4.5. Immunofluorescent Confocal Laser Scanning Microscopy

Imaging on fixed and permeabilized synaptosomes was carried out as previously described [88,92]. The following antibodies were used: rabbit anti-synaptophysin (1:500; Sigma-Aldrich, St. Louis, MO, USA), mouse anti-GFAP (1:1000; Sigma-Aldrich), mouse anti-oligodendrocyte (RIP; 1:10,000; Millipore Corporation) and mouse anti-integrin-αM (1:25; Millipore Corporation). Anti-rabbit and anti-mouse secondary antibodies (conjugated with Alexa Fluor 488 or 633) were used (1:1000; Life Technologies Corporation, Carlsbad, CA, USA). Images were collected by means of confocal microscopy using an inverted Leica STELLARIS 8 Falcon τ-STED (Leica Microsystems, Mannheim, Germany) inverted confocal/STED microscope. A white light laser was used and optimally tuned to provide excitation of the chosen fluorochromes. Hybrid HyD detectors were used for the detection. An HC PL APO CS oil immersion objective 100× (1.40 NA) was used to collect the images, while the pinhole was set to 1 Airy size. The line scanning speed range was 400 Hz. The Leica “LAS X application Suite” software package 4.4.0.24861 was used for acquisition, storage and visualization. The purity of synaptosomal fraction was assessed by analyzing 5–7 fields from at least three different preparations.

### 4.6. Electron Microscopy

For ultrastructural analysis, purified synaptosomes were fixed in 2.5% glutaraldehyde in 0.1M cacodylate buffer, pH 7.2, post-fixed in 1% osmium tetroxide in cacodylate buffer 0.1 M, pH 7.2, en bloc stained with a 1% aqueous solution of uranyl acetate. The dehydration was performed through a graded ethanol series. Samples were then embedded in LX112 (Polysciences Inc., Warrington, PA, USA), polymerized for 12 h at 42 °C, followed by 48 h at 60 °C. A Leica Ultracut E microtome was used to prepare grey-silver ultrathin sections that were then stained with uranyl acetate and lead citrate. All images were acquired using a FEI Talos L120C G2 Transmission Electron Microscope (Thermo Scientific™, Monza, Italy).

### 4.7. Determination of Endogenous Glutamate

The endogenous glutamate released from synaptosomes into the collected superfusate fractions was measured by HPLC, as previously described [87]. The amount of glutamate in the fractions was expressed as pmol/mg protein. The stimulation-evoked glutamate efflux (overflow) was measured by subtracting the estimated basal release from the total amount of glutamate releasaed during and after stimulation. Representative time-courses of glutamate release from the synaptosomes exposed to laser light are reported in Figure 2A.

### 4.8. Calculations and Statistical Analysis

Means ± SEM of the *n* experiments are indicated throughout. Mann–Whitney nonparametric tests or Kruskal–Wallis analysis with multiple comparisons were used to analyze the significance of differences; *p* < 0.05 was taken to indicate statistical significance.

## 5. Conclusions and Perspectives

In conclusion, the ability of near-infrared light to activate glutamatergic transmission might help to elucidate possible mechanisms of PBM therapeutic effects in cognitive impairment or in mood disorders. On the other hand, the power dependency of the glutamate-releasing effects (likely able to cause synaptic glutamate levels from physiologically relevant to potentially neurotoxic) is a key point to be considered when dealing with therapeutic indications for photobiomodulation.

Ultra-weak photon emission, i.e., emission of the so-called biophoton, is hypothesized to be involved in neural signal transmission: it has been reported that glutamate could induce biophoton activity in neuronal circuits [93,94], and that biophotons, possibly transmitted along myelinated axons, may be involved in synaptic plasticity and learning [95,96]. The speculation that biophotons can be involved in neuron communication and repair has been advanced (see [97]). The finding in our experimental conditions that photons can activate vesicular glutamate release from the nerve terminals does not allow for speculation regarding biophotons which are expected to be produced in CNS during glutamatergic transmission activation at a similar wavelength [93,94]. However, our findings might open a new field of investigation on possible mechanisms for neural information transfer via glutamate-evoked biophoton activity, integrating biophoton and neurochemical signaling.

## Data Availability

Data available on request from the corresponding authors.
